# Evaluating Large Language Models for Food Supplement Development: A Case Study in Glycemic Control

**DOI:** 10.3390/nu18081228

**Published:** 2026-04-14

**Authors:** Andor Zsolt Háber, Roland Zsolt Szabó, Mária Figler

**Affiliations:** 1 Doctoral School of Health Sciences, Faculty of Health Science, University of Pécs, 7621 Pécs, Hungary; 2 Department of Corporate Leadership and Marketing, Kautz Gyula Faculty of Business and Economics, Széchenyi István University, 9026 Győr, Hungary; 3 Institute of Nutritional Sciences and Dietetics, Faculty of Health Sciences, University of Pécs, 7621 Pécs, Hungary; 4 2nd Department of Internal Medicine and Nephrology Centre, Clinical Centre, University of Pécs, 7624 Pécs, Hungary

**Keywords:** large language models (LLMs), artificial intelligence in nutrition, food supplement development, glycemic control, prediabetes, nutraceutical formulation, prompt engineering, evidence-based nutrition

## Abstract

**Background/Objectives:** The rapidly expanding landscape of digital technologies is transforming innovation processes across industries, and the food sector is increasingly encouraged to adopt novel tools that can enhance development workflows and support competitive positioning. In the context of Industry 4.0, it is particularly important to examine open innovation approaches that may increase the efficiency of engineers and researchers involved in the research and development of food supplements. Such approaches enable broader access to relevant scientific information, including new bioactive ingredient research and their physiological implications, potentially contributing to the development of better-informed and higher-quality products. **Methods:** In the present study, we evaluated the deep research capabilities of several popular large language models to assess their suitability for supporting the conceptual design of a blood glucose-optimizing food supplement intended for prediabetes management. The comparative analysis focused on the level of detail in the outputs generated by each model, the robustness of the conclusions drawn, and the capacity to produce formulation-oriented recommendations grounded in scientific literature and regulatory frameworks. Our evaluation was primarily qualitative and subjective, highlighting both the potential and limitations of these models. Moreover, the study outlines a forward-looking concept for product validation using wearable smart devices and medically certified wearable devices with continuous biometric monitoring, which could provide an innovative avenue for assessing supplement efficacy. **Results:** The findings indicate that large language models can support the collection, organization, and preliminary interpretation of complex scientific information. **Conclusions:** Nevertheless, expert input remains essential for accurate evaluation, scientific validation, and regulatory compliance, as these models cannot yet replace domain expertise or rigorous experimentation in food supplement development.

## 1. Introduction

From the discovery of vitamins to the present day, the food industry has undergone substantial transformation. The historical development of food supplements (FS) can be traced back more than a century, to Casimir Funk’s 1912 observations, on the basis of which he coined the term “vitamin.” In the decades that followed, numerous vitamins were identified and synthesized, including vitamin C, which remains perhaps the best known to this day [1,2,3]. During the first half of the twentieth century, in response to deficiency diseases such as scurvy, beriberi, and rickets, staple foods began to be fortified with minerals and vitamins as a form of state intervention. Following the successful reduction in deficiency-related disorders, from the 1950s onward, the focus in affluent societies increasingly shifted toward chronic non-communicable diseases associated with modern lifestyles [4]. In an effort to mitigate the risk of such diseases, the concept of functional foods emerged. This term was defined in Japan in the 1980s, with the primary aim of combining physiological benefits with sensory acceptability [5]. Functional foods provide physiological effects beyond their basic nutritional value; however, under European regulations, they are not classified separately from conventional foods. From this developmental trajectory, the category of food supplements subsequently emerged, for which a harmonized European regulatory framework now exists [6]. In the contemporary digital era, and increasingly on the basis of individual characteristics (including formulations aimed at optimizing blood glucose levels), FS have become tools of individualized, personalized health support, with all their associated advantages and potential drawbacks [7].

### 1.1. Digital Transformation and Innovative Approaches in the Food Industry

In the era of Industry 4.0 (IR4.0), alongside the increasing prevalence of civilization-related diseases, growing emphasis is also being placed on global overpopulation and the resulting challenges of sustainable food supply. In addressing these challenges and improving the efficiency of related processes, advanced food industry and healthcare technologies are creating new opportunities, particularly due to the substantial acceleration in the speed of data collection and processing over the past decade [8,9,10,11,12,13]. However, the capabilities of digital tools, including artificial intelligence-based software as a service (SaaS) applications, have not yet been thoroughly investigated in the development of food supplements, particularly regarding their ability to filter, synthesize, and coherently interpret large volumes of scientific data. In addition, the potential of wearable medical devices to monitor and evaluate the effects of specific foods, including FS, remains largely unexplored. These gaps highlight the need for systematic investigation to determine whether digital and AI-based tools can effectively support evidence-based food supplement development and personalized health interventions.

### 1.2. Trends and Dynamics in the European Food Supplement Market

The COVID-19 pandemic further accelerated the adoption of digital technologies and, in parallel, the sale of immune-supporting FS. The food supplement market continues to expand, having grown in Europe by approximately 7–8% in previous years despite global oversupply [14,15,16,17,18]. Product quality also shows considerable variation in international comparison, particularly with regard to novel ingredients, concentration, and compound forms.

In Hungary, more than half of the population regularly purchases at least one type of food supplement [19,20,21]. The number of official notifications also indicates substantial market expansion:; 2777 new products were notified in 2024, while this figure rose to 3435 in 2025 [22]. A similar trend can be observed in Slovakia, where 2214 FS were registered in 2023 and 3286 in 2024 [23]. In both countries, the number of notified products can be considered high relative to population size, which supports the conclusion that the market is expanding intensively and that product supply continues to grow. Capsule formulations dominate the market, presumably due to the more convincing physiological effects that consumers associate with them, as well as their lower excipient content; these are followed by powdered drink formulations and then by products in other dosage forms.

Global economic competitiveness, shortages of skilled labor, variability in raw material quality, new food safety requirements, innovation opportunities, and changing consumer expectations are all forcing food industry companies, on the one hand, to reduce their vulnerability by responding more flexibly to change and, on the other hand, to strengthen their professional and manufacturing advantages, including through improving the efficiency of product development processes.

### 1.3. Research and Development of FS

The development of foods, particularly FS and other functional foods, relies on multidisciplinary expertise involving food engineers, dietitians, chemists, and pharmacists alike. The competencies of these professionals encompass technological design, the selection of active ingredients, the consideration of nutritional physiological and pharmacokinetic aspects, as well as formulation development. In addition, they ensure not only physiological suitability and regulatory compliance, but also contribute to high product quality and consumer satisfaction through the creation of harmonious flavor profiles. Consequently, food development processes often require substantial investments of time and resources [24]. Furthermore, it can be observed that such development still largely takes place within the framework of the classical closed model; consequently, the traditional development process provides a relatively limited scope for the meaningful implementation of open innovation approaches. At the same time, the integration of automated process innovations is not yet common practice, although such approaches could facilitate broader access to emerging knowledge, including information on bioactive ingredients, their potential harmful effects, contraindications, pharmacokinetics, and interactions with other active compounds.

### 1.4. The Role of Artificial Intelligence in the Development Process

The application of artificial intelligence (AI) has already become part of routine practice in both the pharmaceutical and food industries; however, this approach remains less widely recognized in the field of FS development. Emerging digital methodological and technological tools make it possible to improve the efficiency and innovativeness of FS development, for example through the use of generative large language models (LLMs) [25,26,27,28,29,30,31,32,33,34,35,36,37]. These platforms are capable of generating various types of structured digital content on the basis of user instructions. LLMs can efficiently filter, interpret, and summarize large volumes of scientific literature, thereby supporting faster, data-driven, and scientifically grounded decision making [38,39]. Properly designed prompts can enhance the depth of reasoning and the level of detail produced by large language models, thus enabling their effective use even without prior programming expertise [40]. Prompt frameworks developed on the basis of professional considerations (“smart prompt–smart content”) play a key role in ensuring the quality of generated outputs, which is also a determining factor in the development of food supplements [41]. Nevertheless, despite their considerable potential, the application of LLMs is accompanied by important limitations that must be taken into account. These systems may inherit biases from their training data and can also generate new ones due to their underlying algorithms, potentially influencing the interpretation and evaluation of scientific information. Although empirical evidence on such biases remains limited, their existence cannot be excluded; therefore, careful application and further methodological research are required. In addition, the role of LLMs in research evaluation and development processes is still insufficiently explored, with limited empirical validation across different application contexts. Despite these limitations, LLMs remain highly relevant from an innovation perspective, particularly because their ability to access, process, and synthesize external sources of knowledge aligns closely with the principles of open innovation. Open innovation is based on the expansion of knowledge flows, in contrast to traditional innovation models that rely primarily on internal resources. Within this framework, AI-based systems, particularly LLMs, may be interpreted as dynamic and open knowledge integration platforms capable of generating new, complex, and value-added information through the integration of external scientific literature, data sources, and user inputs [42,43]. This form of knowledge transfer may open new opportunities for more data-driven product development. Consequently, while LLMs can serve as powerful supportive tools in FS development, their outputs require critical expert oversight and should be complemented by rigorous scientific validation and domain-specific knowledge.

### 1.5. Relevance of FS in Treatment of Prediabetes

As a consequence of excessive energy intake, unfavorable nutrient composition, particularly high fat and simple sugar consumption, low dietary fiber and micronutrient intake, as well as reduced physical activity, the number of individuals with prediabetes and diabetes is increasing exponentially in both industrialized and developing countries. Prediabetes is a transitional metabolic state situated between normal blood glucose regulation and type 2 diabetes mellitus (T2DM). It is a reversible condition, meaning that the risk of progression to manifest T2DM may be reduced through strict lifestyle modification [44].

Dietary fibers, together with antioxidant bioactive compounds such as polyphenols, including flavonoids, anthocyanins, tannins, and phenolic acids, may play a significant role in the beneficial modulation of glucose metabolism. One important property of polyphenols is their ability to reduce oxidative stress, which plays a key role in pancreatic β-cell dysfunction and in the development of insulin resistance in the pathogenesis of T2DM. By contrast, dietary fibers may contribute to improved glycemic control primarily by slowing carbohydrate absorption, attenuating postprandial blood glucose elevation, and favorably modulating the gut microbiota. In addition, certain bioactive components may influence insulin sensitivity, inflammatory processes, and the signaling pathways involved in the regulation of glucose metabolism. The combined effects of these mechanisms may contribute to lowering blood glucose levels and favorably influencing the prediabetic state; accordingly, these compounds have already been incorporated into numerous functional foods and FS formulations [45,46,47,48,49,50].

The metabolism, absorption, and excretion of bioactive compounds may show substantial interindividual variability, which can directly affect their bioavailability, efficacy, and the potential risk of drug interactions. Since the development and severity of diabetes depend on multiple interrelated factors, the effects of dietary supplements may vary considerably between individuals; therefore, adherence to medical recommendations is of particular importance when such products are used.

### 1.6. Monitoring the Effectiveness of Food Supplements Using Wearable Smart Devices and Biometric Data

Traditionally, the verification of FS efficacy or utilization has been based on laboratory testing. Although this approach provides a general snapshot, it is not capable of capturing the dynamic, day-to-day physiological responses of the body during the use of a given preparation. In recent years, however, wearable medical smart devices have undergone substantial development, together with their ability to provide real-time biometric data. These devices are capable of continuously measuring a range of physiological and biological parameters, including heart rate, heart rate variability (HRV), blood oxygen saturation, blood pressure, skin temperature, sleep quality, stress indicators, and blood glucose levels [51]. The application potential of wearable smart devices is expanding across an increasing number of indications. For example, the effect of a preparation intended to improve sleep quality may be objectively assessed through changes in sleep parameters measured by a wearable smartwatch or ring. Similarly, blood glucose dynamics may be monitored by means of continuous glucose monitoring (CGM). CGM technology enables the minimally invasive, continuous display of overall glucose levels and related indicators, thereby providing information that extends beyond a single fasting blood glucose measurement [52,53,54].

Through the application of CGM, the effects of an FS may, with high probability, be measured objectively and even classified according to efficacy. This makes it possible to assess precisely the extent to which a preparation influences the postprandial glucose curve, fasting blood glucose levels, and glycemic variability relative to the placebo. Importantly, such evaluation can be based not on a single laboratory measurement, but on a continuous data series extending over days or even weeks.

## 2. Materials and Methods

The question was not whether LLMs are capable of generating relevant text, but rather to what depth and with what degree of reliability the generated content can be used in the research and development (R&D) process of an FS. In the present study, the subscription-based deep research functions of six widely used large language models, Google Gemini 3 Pro, OpenAI ChatGPT 5.2 Plus, Perplexity AI Sonar Pro, Grok 4.1, DeepSeek V3.2, and Anthropic Claude Opus 4.6, were compared within the framework of a task aimed at developing a blood glucose-optimizing FS. The responses produced by the models were subjected to comparative analysis in order to examine the extent to which the generated content could be used to support the foundation of a new product development (NPD) process, and to assess the extent to which the models were capable of assisting in such a complex task. Particular attention was paid to the use of the literature references and to the composition of the proposed active ingredient formulations during the evaluation. The results were generated in a single generation cycle without any subsequent modification. All LLMs received the same instruction, “perform the task according to the prompt template.”

### 2.1. Chain-of-Thought Prompt Template

For prompting, we employed a pre-developed template designed to guide the LLM in developing the concept of a premium blood glucose-optimizing food supplement formulation intended to support the metabolic normalization of the prediabetic state and, with regular use, to promote the maintenance of glucose homeostasis. The prompt template was based on a “chain of thought” approach, structured around a systematic internal professional reasoning process, and followed a four-block format. This format provided a thematic framework for large language models to interpret and execute complex product development tasks in a step-by-step manner.

### 2.2. Evaluation of LLMs from a NPD Perspective

The responses generated by the LLMs were compared and evaluated using a predefined set of criteria on a 1–5 scoring scale. To reduce ambiguity and improve interpretability across disciplines, the evaluation criteria were defined in operational terms.

“Scientific depth” referred to the level of mechanistic explanation and integration of relevant scientific knowledge. “Use of sources” captured the presence, relevance, and type of references, while the “quality of references” reflected the scientific credibility and contextual appropriateness of the sources. The level of detail of the “pathophysiological background” indicated the extent to which underlying biological mechanisms were elaborated. “Formulation” was assessed based on the scientific rationale behind ingredient selection, dosing, and combination strategies. The “application of regulatory” considerations evaluated the extent to which legal and safety frameworks were taken into account.

The “document length” was evaluated with the consideration that overly concise responses may lack sufficient scientific detail, thereby limiting their interpretability and applicability. In contrast, more extensive and detailed outputs were regarded as advantageous, as they provide greater depth and broader practical utility. “Practical feasibility” referred to the realism, implementability, and integration potential of the proposed solutions within real-world product development and regulatory environments. The 1–5 scale functioned as a qualitative comparative framework, where lower scores indicated incomplete or less relevant outputs, and higher scores reflected scientifically grounded, coherent, and practically applicable responses. In addition, hallucination phenomena were analyzed separately, with particular attention to fabricated or inaccurate references and claims.

### 2.3. Methodological Limitations

The limitations of the methodology primarily stem from the exploratory nature of the study. The use of a single, standardized prompt ensured comparability across models; however, it did not allow for the assessment of robustness, sensitivity to input variations, or the effects of different prompt formulations. The evaluation was based on expert judgment and was therefore partly subjective, and since the authors themselves conducted the assessment, the potential for evaluator bias cannot be excluded. The applied scoring system served as a comparative framework rather than a validated measurement tool, and due to the complexity of the textual outputs generated, fully quantitative evaluation was only partially feasible. The single-generation approach was intended to model real-world usage scenarios, yet it limits reproducibility, and the lack of external validation further constrains the generalizability of the findings. Taken together, the study provides valuable exploratory insights into the capabilities of LLMs in FS development; however, caution is warranted in interpreting the results, and future research should incorporate independent evaluation, multiple prompt designs, and external validation to enhance robustness and generalizability.

## 3. Results

Based on our findings, it can be concluded that the individual models completed the task with differing levels of effectiveness. The length of the documents generated proved to be informative in itself, as it clearly reflected the strategic approach each model applied to the product development task (Table 1). The most extensive response was generated by Claude AI (42,524 characters), whereas the shortest output was produced by Gemini AI, with a total length of only 4051 characters. Marked differences were also observed in the level of structural elaboration, particularly between outputs resembling “outline-level documentation” and those comparable to “detailed professional summaries.” Claude and Perplexity followed the steps of the prompt template in a systematic manner, employing tables, section references, and internal cross-references, whereas Gemini and Grok addressed the individual steps in short paragraphs of only a few sentences, which limited both internal coherence and the practical usability of the outputs as development documents.

Based on the scoring assessment, Perplexity AI and Claude AI shared first place, each receiving 33 points, and demonstrated broadly similar strengths in both source use and other professional evaluation criteria. Both models applied a detailed and transparent citation system, while also providing well-structured professional summaries of the relevant pathophysiological background. ChatGPT (32 points) stood out from the other models by offering a distinctive formulation proposal and an adequately developed regulatory perspective, although its scientific evidence base was somewhat less comprehensive. DeepSeek (22 points) and Grok (21 points) provided only outline-level starting points, as they fell substantially short in terms of scientific depth. Gemini AI (12 points) presented the weakest concept, delivering a short, summary-style output with minimal use of sources and limited professional depth. Notably, five models received three points for regulatory compliance, suggesting that coverage of the EU/EFSA regulatory framework remains a challenging area for current models, in contrast to the presentation of the pathophysiological background and practical feasibility, where substantially greater variation was observed.

### 3.1. Analysis of the LLMs Citation Capabilities

The six models used a total of 126 citations, from which the unique sources were identified and examined. Among these references, only 4 sources were shared by three models, 10 sources were cited by exactly two models, while the remaining sources appeared in only a single model’s output. This low degree of overlap suggests that, despite receiving the same prompt, the models relied on different scientific source architectures when assembling their content (Table 2).

Claude AI demonstrated the highest number of unique references, including several sources that were not used by any of the other models. Among these, notable examples included a study examining the potential synergistic effects of chromium and biotin, research on the role of vitamin D in the prevention of type 2 diabetes mellitus (T2DM), and an experimental study investigating the genotoxicity of chromium picolinate in a Chinese hamster ovary cell line [69,70,71]. In the case of Claude, as well as some other models, it was also observed that the relevance of certain cited sources was limited in the context of the target task, and in some cases these could have been replaced by more appropriate literature sources [72]. Among Perplexity’s references, the roles of fenugreek, zinc, and vitamin D were particularly emphasized [73,74,75]. ChatGPT’s unique sources included a meta-analysis investigating the effects of myo-inositol, which served as the scientific basis for a distinctive formulation approach [75]. Most of DeepSeek’s references were not conventional academic citations, resulting in the weakest source quality profile among the models. Gemini, due to its very limited number of references, was only of limited evaluative value.

### 3.2. Comparative Overview of Formulation Proposals Across the Tested Models

Comparing the formulations proposed by the examined models represents one of the most tangible dimensions for evaluating product development concepts, as formulation is the stage at which scientific background, regulatory knowledge, and manufacturing feasibility must converge into a single coherent proposal. The formulations suggested by the models can be grouped into two main approaches. The first is a narrower, more “conservative” strategy (4–5 active ingredients), represented by Gemini (chromium picolinate, berberine, cinnamon, and magnesium), Grok (chromium, berberine, ALA, magnesium, and cinnamon), and DeepSeek (berberine, cinnamon, ALA, and chromium). ChatGPT also proposed a narrower core formulation (myo-inositol, cinnamon, magnesium, and chromium), but deliberately reserved berberine and ALA for later product line expansion, reflecting a more cautious regulatory approach. The second is a broader, “multitarget” formulation strategy (9–10 active ingredients), followed by Perplexity (berberine, chromium, cinnamon, ALA, magnesium, zinc, vitamin D3, fenugreek, and banaba) and Claude (berberine, chromium, cinnamon, R-ALA, magnesium, zinc, vitamin D3, banaba, biotin, and black pepper extract) (Table 3).

Berberine was included in the core formulation by the vast majority of the models examined, typically at a daily dose of around 1000 mg. Perplexity, Gemini, and DeepSeek all proposed this dose, while Grok recommended 1500 mg per day divided into three equal doses. This dosage range is broadly consistent with the clinical doses investigated in relevant meta-analyses. At the same time, an important regulatory consideration is that in certain European Union member states, including Hungary, berberine is classified as a botanical ingredient whose use in food supplements is not recommended or is prohibited. ChatGPT was the only model to omit berberine from the core formulation, referring to it only as a possible option for future product line expansion without specifying a concrete dose. In light of the disputed regulatory status of berberine, this approach appears more cautious from a regulatory perspective, although it also limits the therapeutic potential of the formulation.

In the case of chromium picolinate, dose selection also revealed differences that are relevant from a regulatory perspective. Gemini, DeepSeek, and Claude each proposed 400 µg of elemental chromium per day, which exceeds the upper level (UL) permitted by EFSA for use in food supplements. In this context, it is important to note that, according to the World Health Organization’s (WHO) 1996 position statement, supplemental trivalent chromium at a daily dose of 250 µg, taking the average population intake into account, was not considered likely to raise safety concerns, although additional data were deemed necessary to establish a precise safe intake level. Reflecting this precautionary approach, EFSA permits a maximum daily dose of 250 µg chromium in food supplement form; therefore, the recommendations of these three models are problematic from the perspective of regulatory compliance as well [76]. The other models, Perplexity, ChatGPT, and Grok, remained within the daily range of 200 µg, which falls within the permitted limit.

Magnesium was included by almost all models, with daily doses recalculated to elemental magnesium ranging between 100 and 350 mg, most commonly in bisglycinate form, which, according to the clinical literature, provides significantly better bioavailability than oxide or carbonate forms [77]. DeepSeek, however, did not consider magnesium a relevant component and omitted it entirely from the formulation, a decision that is worth reconsidering given the documented high prevalence of magnesium deficiency in the prediabetic population [78].

Alpha-lipoic acid (ALA) was incorporated into the formulations of four models, in the daily dose range of 300–600 mg. Claude and Perplexity explicitly specified the R-enantiomer form (R-ALA), citing its superior bioavailability compared with the racemic mixture [79]. By contrast, ChatGPT and Gemini did not regard ALA as relevant for inclusion in the core formulation. In the case of ChatGPT, this decision, similarly to berberine, was consistent with a strategy of regulatory caution and gradual product line expansion, whereas Gemini did not provide any justification for its omission.

Marked differences were also observed between the narrower and broader formulation approaches with regard to the additional active ingredients. Zinc bisglycinate (15 mg elemental zinc), vitamin D3 (2000 IU), banaba leaf extract, and biotin were included only by Claude and/or Perplexity, while fenugreek extract (300 mg) was proposed exclusively by Perplexity. According to EFSA’s assessment, banaba extract (*Lagerstroemia speciosa*) falls under the Novel Food category; therefore, products containing this ingredient may not be used or placed on the market within the European Union without prior authorization. At the same time, there is a pending health claim related to banaba extract that has not yet been approved, namely that “*L. speciosa* extract helps maintain normal blood glucose levels” and “contributes to normal glucose metabolism.” This further highlights the limitations of the models in processing the relevant legal and regulatory framework [80,81].

Piperine (*Piper nigrum*) (5 mg) was included exclusively by Claude as a bioavailability enhancer. This approach is pharmacologically well founded in the context of improving the low oral bioavailability of berberine and appeared as a unique formulation element among the models examined [82]. Myo-inositol (4000 mg/day) and magnesium citrate were included only in ChatGPT’s formulation, indicating a distinctive conceptual direction that differed from the other models [83].

### 3.3. Packaging Proposals Across the Individual Models

Three distinct approaches emerged with regard to dosage form and product presentation. Gemini, Grok, and DeepSeek developed their formulations exclusively in conventional capsule format: Grok proposed three capsules per day, while both DeepSeek and Gemini recommended two capsules per day. However, issues related to fill weight and manufacturability were either not addressed or discussed only superficially. By contrast, Perplexity and Claude recognized that, given the high number of components in the multitarget formulations and the substantial mass requirements of certain ingredients, particularly magnesium bisglycinate, a capsule format would not be technologically realistic. To address this, Claude also developed a detailed powdered drink formulation as an alternative, using inulin as a prebiotic bulking agent, stevia and erythritol as sweeteners, and natural lemon flavoring. Perplexity similarly favored a powdered drink alternative, with natural flavorings and colorants. ChatGPT, from the outset, conceptualized the product as a stick pack powdered drink, based on the high daily dose requirement of myo-inositol (4 g), which would have been practically unfeasible in capsule form.

### 3.4. Additional Formulation Considerations

The models showed different levels of awareness regarding the selection of excipients. Claude AI explicitly followed a “clean label” concept and provided a detailed rationale for excluding specific excipients. Perplexity AI likewise supplied a detailed excipient list (magnesium stearate, microcrystalline cellulose, and silicon dioxide), supported by manufacturing-related references. ChatGPT explicitly identified ingredients to be avoided as well (for example, maltodextrin and sugar-based carriers), which strengthened the internal consistency of the product profile. As a positive feature, DeepSeek mentioned the exclusion of titanium dioxide and certain artificial sweeteners, although the latter are not relevant in capsule formulations; nevertheless, it justified their exclusion by referring to their potential impact on insulin response. In contrast, Gemini and Grok addressed the issue of excipients only minimally, which does not provide sufficient detail to support a well-founded manufacturing concept. With regard to capsule shell material, Gemini, DeepSeek, and Grok clearly recommended the use of HPMC (hydroxypropyl methylcellulose)-based vegan capsules, which aligns both with premium product positioning and with reaching a broader target population, including vegetarian, vegan, or religiously restricted consumers. DeepSeek additionally raised gelatin-based capsules as an alternative option. The main characteristic features of the individual models are summarized in the following table (Table 4).

## 4. Discussion

It is important to emphasize that the primary aim of the present study was exploratory to investigate the potential and applicability LLMs in the development of FS. The study was not designed as a rigorous experimental or clinical validation but rather as a proof-of-concept and a multi-model comparative exploration, providing insights into the capabilities and limitations of LLMs in supporting product development. The evaluation framework used, while not a formal experimental protocol, ensured comparability across models and consistency in expert judgment. Subjective elements were inherent due to the complexity of the structured textual outputs, which currently cannot be fully assessed quantitatively. Nonetheless, the expert-based and consistent scoring framework allowed for the identification of relevant trends and limitations, offering guidance for future studies with formal validation.

The performance of the individual models differed in both length and content; however, substantial overlaps were also observed in the formulation proposals and in the use of sources. Although differences were identified in the level of professional complexity, the degree of detail, and the interpretation of the regulatory framework, the responses of the models often reflected similar approaches, recurring ingredients, and partly overlapping literature bases. This suggests that the models did not approach the task according to entirely different logics, but rather followed similar “patterns of reasoning” at several points, while differences remained in the level of elaboration and in their professional emphases.

The application potential of LLMs is further strengthened by the fact that, unlike bibliometric indicators, they do not require a multi-year citation “maturation” period and may, therefore, at least in principle, be suitable for evaluating the most recent research outputs. This may be particularly relevant in practical contexts where the rapid interpretation and preliminary quality assessment of the latest scientific evidence are needed [84]. At the same time, however, the interpretation of LLM-generated source use requires careful consideration. It is widely accepted in the literature that citation-based metrics can be regarded only as indirect indicators of research quality, since citations do not reflect scientific value or relevance alone, but may also be influenced by a range of other factors. Nevertheless, highly cited and widely recognized publications often occupy a prominent position in research evaluation, as they are commonly treated as approximate indicators of scholarly significance. This consideration may also be relevant to the interpretation of LLM source use, as such models may tend to prioritize studies that are more visible in bibliometric terms or enjoy greater scientific recognition. The overlap among the cited sources may therefore be explained, at least in part, by the fact that the models identify similar bodies of the literature as scientifically prominent. At the same time, this practice does not guarantee that the selected sources are the most appropriate, the most recent, or the most specific for a given formulation or development objective. The analysis of source citations also indicated that some of the literature used was only partially related to the formulation goal and could have been replaced by studies more directly relevant to food supplement development. Altogether, this suggests that, when evaluating the sources used by LLMs, scientific recognition or citation impact alone should not be regarded as sufficient criteria; the contextual relevance of the cited sources must also be taken into account.

Although the use of references contributed to the emergence of certain distinct formulation approaches, none of the examined models gave sufficient consideration to ingredient-related perspectives that would have been particularly relevant in the context of FS. Such factors include greater emphasis on plant-derived bioactive compounds, including antioxidants and polyphenols, as well as probiotics and dietary fibers, particularly considering that soluble fibers may be more relevant to short-term glycemic control, whereas insoluble fibers may play a more substantial role in longer-term metabolic regulation and antidiabetic effects, thereby representing potentially valuable components in food supplements intended to support blood glucose management [85,86,87,88].

Our research has shown that LLMs were able to generate content with varying levels of performance based on the predefined prompt. At the same time, it became evident that prompt design plays a decisive role in the quality of the outputs and can, to some extent, be adapted to the specific characteristics of individual models, although its exact extent and methodology remain subjects for further research. In our study, a chain-of-thought-style approach proved particularly useful, as it contributed to the generation of more transparent, coherent, and logically structured responses. This suggests that prompting is not merely a technical aid, but rather one of the key factors that substantially influences the practical applicability of LLMs [89].

At the same time, it is important to emphasize that effective performance cannot always be attributed solely to the quality of prompts. In the case of more complex tasks, model adaptability, fine-tunability, and the ability to operate within a more structured execution framework may provide additional benefits. In this context, the collaboration of multiple specialized large language models may further enhance task execution efficiency, particularly in workflows that require diverse reasoning capabilities or multimodal processing. From this perspective, a relevant direction for future research may be the investigation of approaches that complement prompt design with some form of higher-level coordination or task organization logic, thereby supporting more efficient resource utilization and more reliable execution of complex workflows [90].

The limited attention given to these components suggests that the models did not adequately reflect the broader functional ingredient perspective that is often of central importance in the development of innovative product concepts within this sector.

These limitations in source use were also reflected in the formulation proposals themselves. A product development approach can only be regarded as comprehensive if it is supported by a sufficiently diverse and critically selected scientific background, as this may contribute to the development of a more innovative, balanced, and practically relevant product concept. In the present analysis, certain aspects of the literature were represented only partially in the model outputs. For example, safety-related considerations concerning chromium were not consistently reflected in the proposals, suggesting that the interpretation of efficacy in relation to potential formulation risks still requires careful professional judgment. This also underlines that AI-generated proposals may serve as useful starting points during the early stages of concept development, but they do not replace expert evaluation in the product development process.

Among the models examined, ChatGPT was the only one to propose a formulation that did not include berberine, banaba extract, or elevated chromium levels. From the perspective of the current regulatory environment, this may indicate a comparatively more cautious formulation strategy. However, this observation should be interpreted with caution, since regulatory acceptability alone does not automatically imply broader practical applicability or suitability for product development.

If the next objective is to develop or test a product that complies with the current regulatory environment, then, from the perspective of practical applicability, the formulation generated by ChatGPT may represent the most suitable preliminary starting point among the tested models, although its practical applicability would require further expert, regulatory, and experimental validation.

Accordingly, future research should be based on more systematic and rigorously validated approaches, including the development of standardized and reproducible evaluation frameworks to ensure the reliable comparability of different models and prompt strategies. In the longer term, LLMs may contribute to a more evidence-informed, transparent, and efficient development process, particularly when their use is aligned with up-to-date scientific evidence, expert oversight, and applicable regulatory requirements.

## 5. Conclusions

Consumer behavior related to conscious health preservation is undergoing a significant transformation. On the one hand, purchasing decisions remain strongly influenced by advertising and influencers, and, on the other, there is an increasingly pronounced expectation that products should offer an appropriate price-to-value ratio and be supported by scientific evidence. This changing consumer attitude is creating new points of connection between artificial intelligence-based solutions and user expectations, particularly with regard to the speed, transparency, and depth of access to information. Consumers increasingly expect to obtain detailed product-related information quickly, transparently, and with scientific rigor. They also show a growing demand for the communication of the scientific evidence underlying health claims, while actively expanding their own knowledge of functional food ingredients and their health effects. Altogether, this trend highlights the central importance of transparency, credibility, and professionally grounded communication in future food industry innovation.

Within this context, AI may offer new opportunities for FS development to rely more systematically on the most recent scientifically substantiated evidence, while complementing empirical experience, professional expertise, and compliance with the applicable regulatory framework. In this sense, AI integration may be interpreted not only as a technological advancement, but also as a potential shift toward a more evidence-informed development process. Rather than replacing existing professional practices, such approaches may support more efficient decision making and contribute to greater transparency in product development. This may also be relevant to consumer trust, as scientific substantiation and clear communication are important factors in the perceived credibility of products. To strengthen professional acceptance and product credibility, detailed and transparent communication of information remains essential. Although the physical size of product labels limits the amount of text that can be displayed, the inclusion of a QR code may provide consumers with rapid access to a landing page containing relevant background information related to the product, while remaining consistent with regulatory requirements.

CGM technology and wearable biometric smart devices not only expand the toolkit of diabetes management but may also open a new validation dimension for the development of FS, in which efficacy is assessed not through occasional laboratory measurements, but through continuous, individualized physiological data streams. This may lay the foundation for the next stage of development in the FS industry, in which product concepts are shaped not only by the scientific literature and expert knowledge, but also by dynamically generated real-world physiological data. We intend to continue our future research in this direction, with particular emphasis on how product concepts developed with AI-based expert support can be further refined and how their effectiveness may be validated in a clinical setting, initially in the context of a blood glucose-lowering product.

Against this background, the present findings suggest both the promise and the current limitations of LLM-based support. Although it is conceivable that structured prompt templates could be used to encourage LLMs to elaborate complex formulation concepts, including mechanisms of action, dosage recommendations, regulatory compliance, and safety evaluation, the present results indicate that none of the examined models can yet be regarded as fully suitable for the independent and reliable execution of such a task. At the same time, ChatGPT proved to be the most relevant from the perspective of practical applicability.

During the study, we did not assess the trainability of the models, nor did we examine the extent to which the generated content could be further expanded or refined through the use of more precisely formulated prompts. Accordingly, the optimization of prompt templates, as well as the further development and testing of the models in this direction, may constitute the subject of future research.

Overall, it can be concluded that most of the examined models did take into account the fundamental principles of scientifically grounded, evidence-based product development; however, the evaluations they generated did not comprehensively cover the relevant literature. Manual review indicates that, in the field of supporting prediabetes with food supplements, several additional scientifically relevant approaches can be identified, particularly those involving plant-derived antioxidants, polyphenols, and dietary fibers. This suggests that, in its current form, LLM-based support cannot yet be regarded as fully comprehensive and requires further development in order to process the available scientific evidence in a broader and more structured manner.

Furthermore, it should be noted that the dynamic evolution of individual models, their data processing performance, and their deep learning capabilities continue to show significant progress. In parallel, their capacity for transparent handling, interpretability, and processing complex workflows is also gradually improving. Accordingly, an important direction for future research may be the development of approaches that support LLM operation through richer and more reliable knowledge bases, including multi-omics data, curated biomedical databases, and regulatory decision support tools. At the same time, it remains essential that LLMs continue to function as decision support tools under expert supervision, as only this can ensure scientific integrity, consumer safety, and practical applicability. In the longer term, these developmental trends may contribute to more structured and efficient development processes, particularly if such systems become capable of systematically integrating relevant research findings and supporting a deeper interpretation of complex physiological and biochemical relationships. Taken together, this progression may provide a foundation for these models to become increasingly suitable, reliable, and effective tools for supporting such workflows in the future.

## Figures and Tables

**Table 1:** Scored ranking of large language models based on the evaluation criteria applied to the outputs generated from the prompt template (1–5 scale).

Model	Gemini AI	Perplexity AI	ChatGPT	Claude AI	Grok	DeepSeek
Scientific depth	1	4	3	4	3	3
Source use/Quality of references	1	5	4	5	3	3
Number of scientific references	1 (3)	5 (45)	3 (21)	5 (36)	2 (11)	2 (10)
Pathophysiological background	1	4	4	4	2	3
Formulation	2	4	5	4	2	2
Regulatory application	3	3	5	3	3	3
Length of the document (pages)	1 (4)	5 (23)	3 (7)	5 (25)	3 (8)	3 (10)
Based on practical feasibility	2	3	4	3	2	2
Overall score	12	33	32	33	21	22

**Table 2:** Overlap among publications cited by the models.

Reference	Subject Area	Claude	Perplexity	ChatGPT	Gemini	DeepSeek	Grok	Overlap
Akbari et al. (2018) [55]	ALA meta-analysis	✓	✓	–	–	–	✓	3
EFSA Panel on Dietetic Products, Nutrition and Allergies. (2010/2014) [56]	EFSA opinion on chromium	✓	–	–	✓	–	✓	3
Moridpour et al. (2024) [57]	Cinnamon meta-analysis	–	✓	✓	–	–	✓	3
Commission Regulation (EU) No 432/2012 [58]	EU health claims regulation	✓	✓	✓	–	–	–	3
Allen et al. (2013) [59]	Cinnamon systematic review	✓	✓	–	–	–	–	2
Balk et al. (2007) [60]	Chromium meta-analysis	✓	✓	–	–	–	–	2
Judy et al. (2003) [61]	Banaba extract	✓	✓	–	–	–	–	2
Directive 2002/46/EC [62]	Food supplements directive	–	✓	✓	–	–	–	2
Dagogo (2025) [63]	Pathophysiology of prediabetes	–	✓	–	–	✓	–	2
Liang et al. (2019/2021) [64]	Berberine meta-analysis	✓	–	–	–	–	✓	2
Panigrahi.(2023) [65]	Berberine prediabetes RCT	–	✓	–	–	–	✓	2
Veronese et al. (2016/2021) [66]	Magnesium meta-analysis	✓	–	–	–	–	✓	2
Basit et al. (2026) [67]	Magnesium prediabetes meta-analysis	–	✓	✓	–	–	–	2
Zhao et al. (2022) [68]	Chromium T2DM systematic review	–	✓	✓	–	–	–	2

**Table 3:** Comparison of the formulations proposed by the LLMs, with daily doses for each active ingredient.

Active Ingredients	Claude Daily Dose (Stick)	Perplexity Daily Dose (Stick)	ChatGPT Daily Dose (Stick)	Gemini Daily Dose (Capsule)	DeepSeek Daily Dose (Capsule)	Grok Daily Dose (Capsule)
Chromium picolinate	400 µg	200 µg	2 × 100 µg	2 × 200 µg	2 × 200 µg	3 × 67 µg
Berberine HCl	1000 mg	2 × 500 mg	-	2 × 500 mg	2 × 500 mg	3 × 500 mg
Cinnamon extract	250 mg	500 mg	2 × 500 mg	2 × 250 mg	2 × 150–200 mg	3 × 167 mg
Magnesium bisglycinate (elemental)	200 mg	200 mg	-	2 × 175 mg	-	3 × 100 mg
Alpha-lipoic acid	300 mg	300 mg	-	-	2 × 300 mg	3 × 200 mg
Zinc bisglycinate (elemental)	15 mg	15 mg	-	-	-	-
Vitamin D3	2000 IU	2000 IU	-	-	-	-
Banaba leaf extract	32 mg	50 mg	-	-	-	-
Myo-inositol	-	-	2 × 2000 mg	-	-	-
Magnesium citrate (elemental)	-	-	2 × 100 mg	-	-	-
Black pepper extract	5 mg	-	-	-	-	-
Biotin	2500 µg	-	-	-	-	-
Fenugreek extract	-	300 mg	-	-	-	-

**Table 4:** Comparative assessment of the tested LLM outputs across the main evaluation dimensions of the prompt template.

Model	Document Profile	Source Usage	Formulation Concept	Strengths	Limitations	Overall Impression
Gemini AI	Short, concise, mainly summary oriented	Few sources	4-component capsule formula: chromium picolinate, berberine HCl, cinnamon extract, magnesium bisglycinate	Covered the main points; described the indication and basic use logic clearly; included legal/regulatory mentions	Limited scientific depth; few references; the concept and ingredient rationale were only briefly explained	A useful starting outline, but it requires further professional depth
Perplexity AI	Detailed, professionally comprehensive, 23 pages, logically structured	45 APA-style references, mostly PubMed-based, 1 reference could not be verified	9-component daily capsule or drink powder format, multimodal approach	Detailed pathophysiological background; tabulated formula; doses, mechanisms, contraindications, excipients, and monitoring plan included; regulatory section well developed	One inaccurate reference	A strong evidence-based development concept with high professional utility
ChatGPT	Well structured, 7-page document, detailed but mainly summary oriented	21 APA-style references; a large share were regulatory sources, about 8 directly related to active ingredient selection	2 × 1 serving stick pack drink powder: myo-inositol-based formula with cinnamon, magnesium, and chromium	Different product composition, strong handling of technological excipients, avoid list ingredients, usage routine, target population, and market positioning	Scientific evidence base was shallower than the strongest models; literature support for active ingredient selection was less deep	Implementable product concept
Claude AI	Most detailed, 25 pages, highly refined at documentation level	36 APA-style, verifiable and relevant references, legal references handled separately	Complex multi-capsule system; Type A and Type B capsules, alternative drink-powder version included	Deepest professional development; detailed evidence base, synergy and antagonism analysis; Novel Food, safety, usage routine all presented in detail	High complexity and higher daily capsule count	Well structured, comprehensive science-based response
Grok	Short, factual, and minimally detailed	Few references	3-capsules-per-day formula: chromium, berberine, ALA, magnesium, cinnamon	Clear basic indication and target area, simple formulation outline, practical daily use scheme	Limited depth; sparse literature support, market, legal, technological, and mechanistic detail remained underdeveloped	More of a starting sketch than a complete product development document
DeepSeek	Medium length (10 pages), understandable, but less professionally deep	10 references; limited scientific support	Two-capsule formula: berberine, cinnamon bark extract, ALA, chromium picolinate	Clear structure, identified ingredients to avoid, linked the concept to lifestyle; included some marketability considerations	Short justifications, weaker technical terminology, less developed mechanisms and regulatory background	An understandable, medium-detail concept outline

## Data Availability

The original contributions presented in this study are included in the article. Further inquiries can be directed to the corresponding author.

## References

[1] Semba R.D. (2012). The discovery of the vitamins. Int. J. Vitam. Nutr. Res..

[2] Hannus I. (2003). Albert Szent-Györgyi and his life. J. Mol. Struct. THEOCHEM.

[3] Piro A., Tagarelli G., Lagonia P., Tagarelli A., Quattrone A. (2010). Casimir Funk: His discovery of the vitamins and their deficiency disorders. Ann. Nutr. Metab..

[4] Hasler C.M. (2002). Functional foods: Benefits, concerns and challenges—A position paper from the american council on science and health. J. Nutr..

[5] Siró I., Kápolna E., Kápolna B., Lugasi A. (2008). Functional food. Product development, marketing and consumer acceptance—A review. Appetite.

[6] European Parliament and Council (2006). Regulation (EC) No 1924/2006 of the European Parliament and of the Council of 20 December 2006 on nutrition and health claims made on foods. Off. J. Eur. Union.

[7] Rogus S., Lurie P. (2024). Personalized nutrition: Aligning science, regulation, and marketing. Health Aff. Sch..

[8] Kuhl E. (2025). AI for food: Accelerating and democratizing discovery and innovation. npj Sci. Food.

[9] Hassoun A., Aït-Kaddour A., Abu-Mahfouz A.M., Rathod N.B., Bader F., Barba F.J., Biancolillo A., Cropotova J., Galanakis C.M., Jambrak A.R. (2023). The fourth industrial revolution in the food industry—Part I: Industry 4.0 technologies. Crit. Rev. Food Sci. Nutr..

[10] Camaréna S. (2020). Artificial intelligence in the design of the transitions to sustainable food systems. J. Clean. Prod..

[11] Konfo T.R.C., Djouhou F.M.C., Hounhouigan M.H., Dahouenon-Ahoussi E., Avlessi F., Sohounhloue C.K.D. (2023). Recent advances in the use of digital technologies in agri-food processing: A short review. Appl. Food Res..

[12] Thapa A., Nishad S., Biswas D., Roy S. (2023). A comprehensive review on artificial intelligence assisted technologies in food industry. Food Biosci..

[13] Urbán U., Kuruczleki É. (2025). Role of digitalization and digital skills: The case of the agricultural sector. Navigating the Future: Digitalization, Sustainability, and International Business.

[14] Papatesta E.M., Kanellou A., Peppa E., Trichopoulou A. (2023). Is Dietary (Food) Supplement Intake Reported in European National Nutrition Surveys?. Nutrients.

[15] Hamulka J., Jeruszka-Bielak M., Górnicka M., Drywień M.E., Zielinska-Pukos M.A. (2020). Dietary Supplements during COVID-19 Outbreak. Results of Google Trends Analysis Supported by PLifeCOVID-19 Online Studies. Nutrients.

[16] Hassoun A., Bekhit A.E.-D., Režek Jambrak A., Regenstein J.M., Chemat F., Morton J.D., Gudjónsdóttir M., Carpena M., Prieto M.A., Varela P. (2024). The fourth industrial revolution in the food industry—Part II: Emerging food trends. Crit. Rev. Food Sci. Nutr..

[17] MarketsandMarkets (n.d.). Europe Dietary Supplements Market. https://www.marketsandmarkets.com/Market-Reports/europe-dietary-supplements-market-246220087.html.

[18] Market Data Forecast (2024). Europe Dietary Supplements Market. https://www.marketdataforecast.com/market-reports/europe-dietary-supplements-market.

[19] Nábrádi Zs Bánáti D., Szakály Z. (2020). A study on consumer habits in the dietary supplements market. Appl. Stud. Agribus. Commer.—APSTRACT.

[20] Bilia A.R. (2015). Herbal medicinal products versus botanical-food supplements in the European market: State of art and perspectives. Nat. Product. Commun..

[21] Ransley J.K., Ransley J.K., Donnelly J.K., Read N.W. (2001). The rise and rise of food and nutritional supplements—An overview of the market. Food and Nutritional Supplements.

[22] National Institute of Pharmacy and Nutrition (OGYÉI) (2025). List of Notified Dietary Supplements (2004–2026.02.28). https://ogyei.gov.hu/ETREND_LISTA/.

[23] Public Health Authority of the Slovak Republic (n.d.). Registration of Food Supplements. https://www.uvzsr.sk/web/uvzen/registration-of-food-supplement.

[24] Nnadiegubulam J.C., Harbourne N., Grasso S. (2025). Co-creation in new food development: Current trends, challenges, and future directions. Future Foods.

[25] Alasi S.O., Sanusi M.S., Sunmonu M.O., Odewole M.M., Adepoju A.L. (2024). Exploring recent developments in novel technologies and AI integration for plant-based protein functionality: A review. J. Agric. Food Res..

[26] Biswas T. (2024). Exploring the future of Artificial Intelligence in recipe development: A preliminary study. Int. J. Artif. Intell. Res. Dev. (IJAIRD).

[27] Chang J., Wang H., Su W., He X., Tan M. (2025). Artificial intelligence in food bioactive peptides screening: Recent advances and future prospects. Trends Food Sci. Technol..

[28] Chauhan S., Kerr A., Keogh B., Nolan S., Casey R., Adelfio A., Murphy N., Doherty A., Davis H., Wall A.M. (2021). An Artificial-Intelligence-Discovered Functional Ingredient, NRT_N0G5IJ, Derived from Pisum sativum, Decreases HbA1c in a Prediabetic Population. Nutrients.

[29] Cui Z., Qi C., Zhou T., Yu Y., Wang Y., Zhang Z., Zhang Y., Wang W., Liu Y. (2024). Artificial intelligence and food flavor: How AI models are shaping the future and revolutionary technologies for flavor food development. Compr. Rev. Food Sci. Food Saf..

[30] David L., Thakkar A., Mercado R., Engkvist O. (2020). Molecular representations in AI-driven drug discovery: A review and practical guide. J. Cheminform..

[31] Doherty A., Wall A., Khalid N., Kussmann M. (2021). Artificial Intelligence in Functional Food Ingredient Discovery and Characterisation: A Focus on Bioactive Plant and Food Peptides. Front. Genet..

[32] Herrera-Rocha F., Fernández-Niño M., Duitama J., Cala M.P., Chica M.J., Wessjohann L.A., Davari M.D., Barrios A.F.G. (2024). FlavorMiner: A machine learning platform for extracting molecular flavor profiles from structural data. J. Cheminform..

[33] K onfo T.R.C., Koudoro A.Y., Tchekessi C.K.C., Chadare F.J., Avlessi F., Sohounhloue C.K.D. (2025). Harnessing artificial intelligence for the analysis of complex chemical combinations, paving the way for novel flavors in food manufacturing: A comprehensive review. Food Chem. Adv..

[34] Mak K.-K., Pichika M.R. (2019). Artificial intelligence in drug development: Present status and future prospects. Drug Discov. Today.

[35] Van der Lee M., Swen J.J. (2023). Artificial intelligence in pharmacology research and practice. Clin. Transl. Sci..

[36] Pennells J., Watkins P., Bowler A.L., Watson N.J., Knoerzer K. (2025). Mapping the AI Landscape in Food Science and Engineering: A Bibliometric Analysis Enhanced with Interactive Digital Tools and Company Case Studies. Food Eng. Rev..

[37] Chalasani S.H., Syed J., Ramesh M., Patil V., Pramod Kumar T.M. (2023). Artificial intelligence in the field of pharmacy practice: A literature review. Explor. Res. Clin. Soc. Pharm..

[38] Ma P., Tsai S., He Y., Jia X., Zhen D., Yu N., Wang Q., Ahuja J.K.C., Wei C.-I. (2024). Large language models in food science: Innovations, applications, and future. Trends Food Sci. Technol..

[39] Abdurahman S., Ziabari A.S., Moore A.K., Bartels D.M., Dehghani M. (2025). A primer for evaluating large language models in social-science research. Adv. Methods Pract. Psychol. Sci..

[40] White J., Fu Q., Hays S., Sandborn M., Olea C., Gilbert H., Elnashar A., Spencer-Smith J., Schmidt D.C. (2023). A Prompt Pattern Catalog to Enhance Prompt Engineering with ChatGPT. arXiv.

[41] Luo F., Zhang J., Wang Q., Yang C. (2025). Leveraging Prompt Engineering in Large Language Models for Accelerating Chemical Research. ACS Cent. Sci..

[42] Chesbrough H.W. (2003). The open-innovation model. MIT Sloan Manag. Rev..

[43] Filieri R. (2013). Consumer co-creation and new product development: A case study in the food industry. Mark. Intell. Plan..

[44] Hossain M.J., Al-Mamun M., Islam M.R. (2024). Diabetes mellitus, the fastest growing global public health concern: Early detection should be focused. Health Sci. Rep..

[45] Yuksek E.N., Pereira A.G., Prieto M.A. (2025). Dietary Supplements Derived from Food By-Products for the Management of Diabetes Mellitus. Antioxidants.

[46] Dawi J., Misakyan Y., Affa S., Kades S., Narasimhan A., Hajjar F., Besser M., Tumanyan K., Venketaraman V. (2024). Oxidative Stress, Glutathione Insufficiency, and Inflammatory Pathways in Type 2 Diabetes Mellitus: Implications for Therapeutic Interventions. Biomedicines.

[47] Viana M.D.M., Santos S.S., Cruz A.B.O., de Jesus M.V.A.C., Lauria P.S.S., Lins M.P., Villarreal C.F. (2025). Probiotics as Antioxidant Strategy for Managing Diabetes Mellitus and Its Complications. Antioxidants.

[48] Shahwan M., Alhumaydhi F., Ashraf G.M., Hasan P.M.Z., Shamsi A. (2022). Role of polyphenols in combating Type 2 Diabetes and insulin resistance. Int. J. Biol. Macromol..

[49] Clemente-Suárez V.J., Martín-Rodríguez A., Beltrán-Velasco A.I., Rubio-Zarapuz A., Martínez-Guardado I., Valcárcel-Martín R., Tornero-Aguilera J.F. (2025). Functional and Therapeutic Roles of Plant-Derived Antioxidants in Type 2 Diabetes Mellitus: Mechanisms, Challenges, and Considerations for Special Populations. Antioxidants.

[50] Tobias D.K., Ley S.H., Bhupathiraju S.N., Li L.J., Chavarro J.E., Sun Q., Hu F.B., Zhang C. (2021). Prepregnancy plant-based diets and the risk of gestational diabetes mellitus: A prospective cohort study of 14,926 women. Am. J. Clin. Nutr..

[51] Del-Valle-Soto C., Briseño R.A., Valdivia L.J., Nolazco-Flores J.A. (2024). Unveiling wearables: Exploring the global landscape of biometric applications and vital signs and behavioral impact. BioData Min..

[52] Holzer R., Bloch W., Brinkmann C. (2022). Continuous Glucose Monitoring in Healthy Adults-Possible Applications in Health Care, Wellness, and Sports. Sensors.

[53] Mansour M., Darweesh M.S., Soltan A. (2024). Wearable devices for glucose monitoring: A review of state-of-the-art technologies and emerging trends. Alex. Eng. J..

[54] Zahalka S.J., Galindo R.J., Shah V.N., Low Wang C.C. (2024). Continuous Glucose Monitoring for Prediabetes: What Are the Best Metrics?. J. Diabetes Sci. Technol..

[55] Akbari M., Ostadmohammadi V., Lankarani K.B., Tabrizi R., Kolahdooz F., Khatibi S.R., Asemi Z. (2018). The effects of alpha-lipoic acid supplementation on glucose control and lipid profiles among patients with metabolic diseases. Metabolism.

[56] (2014). EFSA Panel on Dietetic Products, Nutrition and Allergies. Scientific Opinion on Dietary Reference Values for chromium. EFSA J..

[57] Moridpour A.H., Kavyani Z., Khosravi S., Farmani E., Daneshvar M., Musazadeh V., Faghfouri A.H. (2024). The effect of cinnamon supplementation on glycemic control in T2DM: An updated systematic review and dose-response meta-analysis. Phytother. Res..

[58] European Union (2012). Commission Regulation (EU) No 432/2012 establishing a list of permitted health claims made on foods. Off. J. Eur. Union.

[59] Allen R.W., Schwartzman E., Baker W.L., Coleman C.I., Phung O.J. (2013). Cinnamon use in type 2 diabetes: An updated systematic review and meta-analysis. Ann. Fam. Med..

[60] Balk E.M., Tatsioni A., Lichtenstein A.H., Lau J., Pittas A.G. (2007). Effect of chromium supplementation on glucose metabolism and lipids: A systematic review of randomized controlled trials. Diabetes Care.

[61] Judy W.V., Hari S.P., Stogsdill W.W., Judy J.S., Naguib Y.M., Passwater R. (2003). Antidiabetic activity of a standardized extract (Glucosol) from Lagerstroemia speciosa leaves in Type II diabetics: A dose-dependence study. J. Ethnopharmacol..

[62] (2002). Directive 2002/46/EC of the European Parliament and of the Council of 10 June 2002 on the approximation of the laws of the Member States relating to food supplements. Off. J. Eur. Union.

[63] Dagogo-Jack S. (2025). Pathobiology of Prediabetes: Understanding and Interrupting Progressive Dysglycemia. Diabetes.

[64] Liang Y., Xu X., Yin M., Zhang Y., Huang L., Chen R., Ni J. (2019). Effects of berberine on blood glucose: A systematic review and meta-analysis. Endocr. J./JBBS.

[65] Panigrahi A., Mohanty S. (2023). Efficacy and safety of HIMABERB Berberine on glycemic control in patients with prediabetes. BMC Endocr. Disord..

[66] Veronese N., Watutantrige-Fernando S., Luchini C., Solmi M., Sartore G., Sergi G., Manzato E., Barbagallo M., Maggi S., Stubbs B. (2016). Effect of magnesium supplementation on glucose metabolism in people with or at risk of diabetes: A systematic review and meta-analysis of double-blind randomized controlled trials. Eur. J. Clin. Nutr..

[67] Basit A., Kumar S., Ahmed H., Babar R., Saeed S.S., Siddiqui T.A., Khan S., Saeed A., Khan M., Hanif H. (2026). Impact of oral magnesium supplementation on glycemic and cardiometabolic outcomes in prediabetic adults. J. Diabetes Metab. Disord..

[68] Zhao F., Pan D., Wang N., Xia H., Zhang H., Wang S., Sun G. (2022). Effect of Chromium Supplementation on Blood Glucose and Lipid Levels in Patients with Type 2 Diabetes Mellitus: A Systematic Review and Meta-Analysis. Biol. Trace Elem. Res..

[69] Albarracin C.A., Fuqua B.C., Evans J.L., Goldfine I.D. (2008). Chromium picolinate and biotin combination improves glycemic control in people with type 2 diabetes mellitus: A placebo-controlled, double-blinded, randomized clinical trial. Diabetes/Metab. Res. Rev..

[70] Stearns D.M., Wise JPSr Patierno S.R., Wetterhahn K.E. (1995). Chromium(III) picolinate produces chromosome damage in Chinese hamster ovary cells. FASEB J..

[71] Pittas A.G., Dawson-Hughes B., Sheehan P., Ware J.H., Knowler W.C., Aroda V.R., Brodsky I., Ceglia L., Chadha C., Chatterjee R. (2019). Vitamin D Supplementation and Prevention of Type 2 Diabetes. N. Engl. J. Med..

[72] Knowler W.C., Barrett-Connor E., Fowler S.E., Hamman R.F., Lachin J.M., Walker E.A., Nathan D.M., Diabetes Prevention Program Research Group (2002). Reduction in the incidence of type 2 diabetes with lifestyle intervention or metformin. N. Engl. J. Med..

[73] Shabil M., Bushi G., Bodige P.K., Maradi P.S., Patra B.P., Padhi B.K., Khubchandani J. (2023). Effect of Fenugreek on Hyperglycemia: A Systematic Review and Meta-Analysis. Medicina.

[74] Kim J., Noh W., Kim A., Choi Y., Kim Y.S. (2023). The Effect of Fenugreek in Type 2 Diabetes and Prediabetes. Nutrients.

[75] Ranasinghe P., Wathurapatha W.S., Galappatthy P., Katulanda P., Jayawardena R., Constantine G.R. (2018). Zinc supplementation in prediabetes: A randomized double-blind placebo-controlled clinical trial. J. Diabetes.

[76] Hungarian National Institute of Pharmacy and Nutrition (OGYÉI) (n.d.). Vitamins and Minerals Permitted for Use in Food Supplements. https://ogyei.gov.hu/etrend_kiegeszitokben_felhasznalhato_vitaminok_es_asvanyi_anyagok.

[77] Schuette S.A., Lashner B.A., Janghorbani M. (1994). Bioavailability of magnesium diglycinate vs magnesium oxide in patients with ileal resection. JPEN J. Parenter. Enteral Nutr..

[78] Salehidoost R., Taghipour Boroujeni G., Feizi A., Aminorroaya A., Amini M. (2022). Effect of oral magnesium supplement on cardiometabolic markers in people with prediabetes: A double blind randomized controlled clinical trial. Sci. Rep..

[79] Salehi B., Berkay Yılmaz Y., Antika G., Boyunegmez Tumer T., Fawzi Mahomoodally M., Lobine D., Akram M., Riaz M., Capanoglu E., Sharopov F. (2019). Insights on the Use of α-Lipoic Acid for Therapeutic Purposes. Biomolecules.

[80] Mirhosseini N., Vatanparast H., Mazidi M., Kimball S.M. (2018). Vitamin D Supplementation, Glycemic Control, and Insulin Resistance in Prediabetics: A Meta-Analysis. J. Endocr. Soc..

[81] European Commission (2024). Notification 2024.3449: Unauthorised Novel Food Ingredient … RASFF Window. https://webgate.ec.europa.eu/rasff-window/screen/notification/682100.

[82] Tripathi D., Gupta V.K., Pandey P., Rajinikanth P.S. (2025). Metabolic Insights into Drug Absorption: Unveiling Piperine’s Transformative Bioenhancing Potential. Pharm. Res..

[83] Miñambres I., Cuixart G., Gonçalves A., Corcoy R. (2019). Effects of inositol on glucose homeostasis: Systematic review and meta-analysis of randomized controlled trials. Clin. Nutr..

[84] Thelwall M. (2025). Research quality evaluation by AI in the era of large language models: Advantages, disadvantages, and systemic effects—An opinion paper. Scientometrics.

[85] Gowd V., Xie L., Zheng X., Chen W. (2019). Dietary fibers as emerging nutritional factors against diabetes: Focus on the involvement of gut microbiota. Crit. Rev. Biotechnol..

[86] Kaczmarczyk M.M., Miller M.J., Freund G.G. (2012). The health benefits of dietary fiber: Beyond the usual suspects of type 2 diabetes mellitus, cardiovascular disease and colon cancer. Metabolism.

[87] Razmpoosh E., Javadi M., Ejtahed H.S., Mirmiran P. (2016). Probiotics as beneficial agents in the management of diabetes mellitus: A systematic review. Diabetes Metab. Res. Rev..

[88] Naz R., Saqib F., Awadallah S., Wahid M., Latif M.F., Iqbal I., Mubarak M.S. (2023). Food Polyphenols and Type II Diabetes Mellitus: Pharmacology and Mechanisms. Molecules.

[89] Al Shuraiqi S., AlZaabi A., Aal Abdulsalam A. (2026). Prompt Engineering Strategies for Generating Medical Case-Based MCQs with Large Language Models: A Multi-Model Comparative Study. Mach. Learn. Knowl. Extr..

[90] Luo H., Liu Y., Zhang R., Wang J., Sun G., Niyato D., Yu H., Xiong Z., Wang X., Shen X. (2025). Toward Edge General Intelligence with Multiple-Large Language Model (Multi-LLM): Architecture, Trust, and Orchestration. arXiv.

